# A Loss Cycle of Burnout Symptoms and Reduced Coping Self-Efficacy: A Latent Change Score Modelling Approach

**DOI:** 10.1177/24705470241286948

**Published:** 2024-10-07

**Authors:** Liselotte M.J. Koning-Eikenhout, Roos Delahaij, Wim Kamphuis, Inge L. Hulshof, Joris Van Ruysseveldt

**Affiliations:** 1 Faculty of Psychology, 10198 Open Universiteit, Heerlen, the Netherlands; 2 Learning & Workforce Development, 312992 TNO Netherlands Organisation for Applied Scientific Research, Soesterberg, the Netherlands

**Keywords:** resources, depletion, loss cycle, coping self-efficacy, burnout, Latent Change Score, police officers, efficacy beliefs, Conservation of Cesources, Job Demands-Resources

## Abstract

Police officers are frequently faced with chronic and acute stressors, such as excessive workload, organizational stressors and emotionally charged reports. This study aims to examine the relationship between a form of chronic strain (ie, burnout symptoms) and a resource (ie, coping self-efficacy) in a sample of Dutch police officers. Specifically, we aim to investigate the existence of a loss cycle of resources. We use Latent Change Score modeling to investigate the potential depletion or loss cycle of coping self-efficacy as a result of burnout symptoms in a sample of 95 police officers who completed a survey on three consecutive timepoints. The lag between the measurements was approximately one year. We found that, during both one-year intervals, within-person increases in burnout symptoms were related to within-person decreases in coping self-efficacy. Also, the results emphasize the buffering role of coping self-efficacy for burnout symptoms, as within-person decreases in coping self-efficacy during the first year were associated with within-person increases in burnout symptoms during the following year. Together, the results imply that a loss cycle of coping self-efficacy and burnout symptoms may occur. For this we used Latent Change Score modeling, which is a relatively new approach which provides researchers with the opportunity to analyse multi-wave longitudinal data while focusing on within-person changes over time. Practically, police organizations are advised to monitor personnel wellbeing and resources, to maintain and promote sustainable employability of police officers and to be able to timely provide individuals with interventions. Limitations discussed are the use of self-report measures and large intervals between the measurements. Finally, future directions of research are discussed that would circumvent the reported limitations, such as multiple wave with shorter lags and incorporating confounding factors that could affect coping self-efficacy.

## Introduction

Police officers frequently encounter chronic and acute stressors. Coping self-efficacy, that is someone's belief in their ability to deal with stressors, is an especially crucial resource for police officers and other high-risk professionals, as it can act as a buffer in the stressor-strain relationship. It may help prevent or mitigate stress-related complaints such as psychological and health problems. For example, Delahaij et al showed that during military deployment, in cases of threatening situations, service members’ strong self-efficacy led to more work engagement and less burnout symptoms.^1^ Another study showed that low coping self-efficacy before deployment and decreasing coping self-efficacy over time are related to the development of post-traumatic stress disorder symptoms 5 years after deployment.^2^ Also, Eikenhout et al showed that police officers with high coping self-efficacy experience less burnout symptoms from organizational stressors such as scheduling issues and interruptions.^3^

Generally, research focuses on the buffering effect of coping self-efficacy as a personal resource. To our knowledge, limited research has been done on coping self-efficacy as an outcome of the stress process. However, in line with the Conservation of Resources (COR) theory,^4^ coping self-efficacy can be considered a result of mastery of stressful circumstances, and thus can be depleted by a lack of mastery or capability to overcome stress.^5^ Some examples exist of research investigating efficacy or efficacy beliefs as outcomes. For instance, Llorens and colleagues investigated task resources as positive outcomes of work engagement and found that engagement increases future efficacy beliefs, and lies at the core of those beliefs.^6^ Furthermore, Llorens-Gumbau and Salanova-Soria showed an energy depletion process for teachers, where exhaustion and cynicism because of work obstacles, led to a decrease in self-efficacy.^7^ Also, Eikenhout et al showed that police officers who experienced burnout symptoms in one year, had lower coping self-efficacy in the next year.^3^ Although that study focused on a chronic stress process with fixed measures of burnout and coping self-efficacy, results implied that depletion of coping self-efficacy is plausible.^3^ At the time of writing, we have not found literature describing a within-person depletion effect of changes in burnout on changes in coping self-efficacy in police officers.

Research into a depletion of coping resources is important for two reasons. First, because coping self-efficacy as a predictor is important in preventing future negative outcomes, it is crucial to investigate what factors may influence such coping self-efficacy. Second, investigating depletion of resources is crucial for police officers, as psychological and physiological symptoms stemming from stress may lead to long-term absenteeism and turnover. An outflow of personnel may result in a decline of police capacity, which is reaching its lower limits in the Netherlands. For example, in the years to come, the Netherlands police is supposed to increase the force with 17.000 new police officers on top of the existing 62.000 in 2021.^8^ According to the latest national survey of employes 17.3% of security workers (of which police is one profession) experienced burnout-related complaints such as exhaustion due to work, while 2.8% of respondents in the same profession have reported that they have officially been diagnosed with being overworked or having a burnout.^9,10^ Investigating a potential loss cycle of resources in police officers is another step towards diminishing the risk of burnout and promoting sustainable employability among police personnel.

This research contributes to the literature in two ways. In contrast to previous research investigating the relationship between resources as a buffer and burnout as an outcome, we focus on the potential depletion of a resource (ie, coping self-efficacy) due to burnout symptoms. Moreover, in this study we use Latent Change Score (LCS) modeling to investigate this effect. This approach provides the opportunity to analyse multi-wave longitudinal data while focusing on within-person changes over time. That is, using the strength of Structural Equation Modelling (SEM), LCS provides an important, relatively new approach to analyse longitudinal data with a focus on changes within persons.^11,12^ With LCS, we investigate the within-person effects of changes in burnout on changes in coping self-efficacy during the same unit of time. Also, a lagged effect of changes in coping self-efficacy on changes in burnout will be investigated to examine whether the depletion of resources reinforces the expected negative cycle.

### Theoretical Background

COR theory^4,13^ describes the human drive to protect, maintain and gather resources. Furthermore, it states that individuals rely on resources to prevent strain and that they experience strain when these resources are lost or threatened. The Job Demands-Resources (JD-R) model supports this, as it proposes that resources can have a direct effect on motivational outcomes (eg, job satisfaction) and can act as a buffer to prevent negative health outcomes (eg, burnout).^14^ Research using the JD-R model has confirmed that resources can indeed moderate the relationship between stressors and for example burnout.^1,3,15–17^ When resources are lost, these resources are not available to prevent future strain or to invest for future resource gain. Hobfoll and colleagues describe this process as a loss spiral, where loss begets future loss.^13^ So, having limited resources makes an individual vulnerable to future resource loss and less capable of resource gain. Therefore, we believe it is important to investigate whether resources can be depleted as a result of chronic strain, in addition to the buffering effect of resources on chronic strain.

### Coping Self-Efficacy as a Predictor of Burnout

Efficacy beliefs are a personal resource that can develop from several sources, like affective states and earlier coping experiences.^18,19^ “Perceived self-efficacy refers to the beliefs in one's capabilities to organize and execute the courses of action required to produce given attainments.”^5^ Coping self-efficacy is then defined as the self-appraised capability to manage stressful or threatening situations.^20^ Accordingly, coping self-efficacy includes concepts such as beliefs on stress resilience, stress recovery, and (police) task-efficacy. As mentioned before, coping self-efficacy can buffer the relationship between stressors and chronic strain.^3^ Burnout symptoms are a form of chronic strain. It is defined as a slow-developing, long-term, work-related condition.^21^ It is mainly characterized by exhaustion, dysregulation of cognitive and emotional processes, and mental distancing.^21,22^ Coping resources (ie, coping self-efficacy) and burnout are related,^5^ because burnout develops when demands exceed the ability to cope effectively (ie, when demands and resources are out of balance).^23^

### Burnout as a Predictor of Coping Self-Efficacy

The mechanism through which burnout symptoms can lead to resource loss is twofold. On the one hand, the onset of burnout symptoms can be considered the consequence of a lack of mastery of coping with stress. So, it can be considered the result of negative experiences with an inability to effectively cope with stressors. This lack of mastery degrades efficacy beliefs (vs mastery experiences that can enhance efficacy beliefs).^5^ In fact, early models of burnout included reduced personal accomplishment as a key dimension, which referred to feelings of incompetence and a lack of achievement.^21,24^ On the other hand, burnout is characterized by exhaustion accompanied by negative affect and attitudes towards oneself and others.^25^ According to Bandura, affective states can influence perceived self-efficacy.^5^ According to Schwartz and Clore, evaluative judgement can be influenced by the perceived informative value of affective states.^in^^5^ That is, people make positive evaluations when they are experiencing positive emotions (positive state), and negative evaluations when they are experiencing negative emotions (negative state). In turn, these emotions and evaluations can enhance or diminish self-appraisal and perceived efficacy.^5^ Together, this suggests that burnout symptoms, as an instigator of negative affective states and as negative experiences with coping, can negatively affect efficacy beliefs and the availability of resources for future coping.

## Research Question and Hypotheses

**Figure 1.**
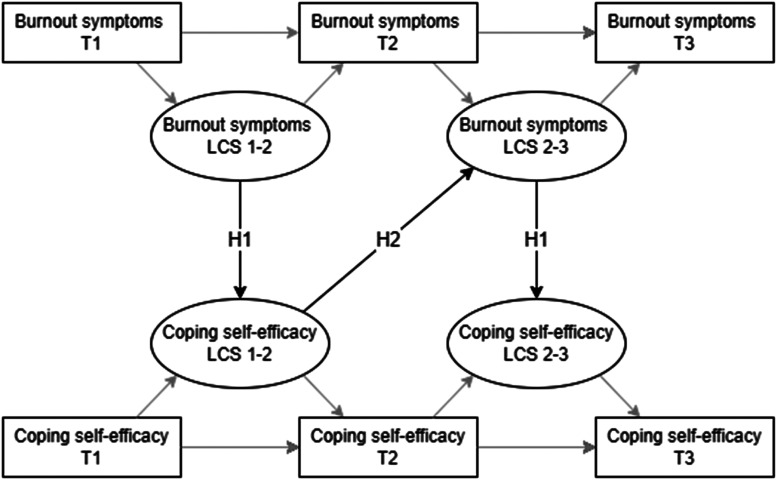
Research model with the hypothesized relationships. T = time, LCS 1-2 = Latent Change Score from time 1 to time 2, LCS 2-3 = Latent Change Score from time 2 to time 3.

This study aims to examine the temporal relationship between burnout and coping self-efficacy in a sample of Dutch police officers. To investigate the potential depletion of coping self-efficacy as a result of burnout symptoms, the present study builds on a previously obtained dataset that included three-waves of these variables.^viz.^^3^ Unlike the original article using this dataset, we use a LCS modeling approach to zoom in on within-person relationships. Between-person analyses allow researchers to investigate differences between individuals. On the other hand, within-person analyses allow researchers to draw conclusions regarding intra-individual mechanisms, in this case the mechanism of depleting personal resources or developing burnout symptoms over time. In addition, adding lagged effects (ie, relationship between change scores of succeeding periods) allows researchers to more clearly establish causal relationships between variables (ie, coping self-efficacy and burnout symptoms) within individuals. Focusing on within-person changes over time may further enhance the understanding of the impact of burnout symptoms on coping self-efficacy, which may provide organizations with important insight for developing effective interventions. Based on the concept of loss cycle of resources,^4^ theory on efficacy beliefs,^5^ and prior research into the buffering effect of coping self-efficacy against burnout,^14^ we hypothesized that a loss cycle of burnout and coping self-efficacy exists. That is, an increase in burnout symptoms can lead to a decrease in coping self-efficacy during the same year (H1). In turn, this decrease in coping self-efficacy can undermine its buffering effect and future coping efficacy, resulting in an increase in burnout symptoms the next year (H2). This increase in burnout symptoms would yet again lead to a decrease in coping self-efficacy (H1), perpetuating a loss cycle (Figure 1):

**H1:** Within a one-year period, increases in burnout symptoms are related to decreases in coping-self-efficacy.
**H2:** One-year decreases in coping self-efficacy predict increases in burnout symptoms in the following year.

## Methods

### Participants and Procedure

This article uses a dataset previously published in another article.^viz.^^3^ For this study, a three-wave questionnaire was administered online at the police district of a large city in the Netherlands. Data collection took place in May 2019 (T1), March 2020 (T2), and March 2021 (T3). This study was approved by an internal ethical committee (registration number: 2018-075): Research participants were treated in accordance with the ethical guidelines set out by the American Psychological Association.^26^ Participants were tracked over time with pseudonyms. Participants received information on the questionnaire's process, goals, anonymity, and confidentiality. Subsequently, participants gave their informed consent by actively ticking a box to begin the questionnaire.

For the first wave, police officers were approached through flyers and human resources. Participants registered through a designated email address. For waves 2 and 3, police officers were approached directly via email. To increase the response rate, several reminders were sent. Only participants who completed three consecutive waves were included in this study, which resulted in a sample of 95 participants. The majority of the sample were over 30 years old (49% between 30 and 45; 45% older than 45 years). Most police officers had received vocational education (44%; 29% higher education; 27% high school). Gender was not registered to reduce traceability to individuals within their teams.

### Measures

In contrast to generalized efficacy measures, tailored efficacy measures are stronger predictors of person- and situation-specific behavior and outcomes.^5^ Since the focus of this study was to investigate burnout symptoms and coping self-efficacy in the police context, we measured coping self-efficacy. Coping self-efficacy was measured with 10 items from the Police Resilience Monitor.^27^ The items are related to stress resiliency, stress recovery, and police task-efficacy. Examples items are “I'm good at dealing with unpleasant feelings” and “I trust my own skills as a police officer.” Cronbach's alpha was .85 (T1), .89 (T2), and .89 (T3).

Burnout was measured with 10 items: It included five items for emotional exhaustion from the National Working Conditions Survey, which is based on the Utrecht Burnout Scale,^28^ and five items for depersonalization from the Utrecht Burnout Scale for Contact Professions.^29^ Answers were rated on a seven-point scale, ranging from 1 (never) to 7 (every day). Example statements are “I feel completely exhausted because of my work” and “I do not really care about what happens to some people.” Cronbach's alpha was .87 (T1), .89 (T2), and .92 (T3).

### Statistical Analysis

The data was analysed using an LCS approach. With SPSS Amos (Statistical Package for the Social Sciences - Analysis of Moment Structures) 24, SEM (Structural Equation Modelling) was applied to investigate the research model. LCSs were calculated for both the predictor (burnout symptoms) and the outcome (coping self-efficacy) to investigate our hypotheses with the research model. This allows us to investigate within-person changes over time, while taking into account the repetition of measurements (ie, using the same measurement at T1, T2, and T3).

To assess the fit of the empirical model, we examined different fit indices (eg Kline, 2005): the Chi Square (χ2), the comparative fit index (CFI), the normed fit index (NFI), the Tucker–Lewis index (TLI) and the root mean squared error of approximation (RMSEA). Values above .90 for the CFI and the TLI are considered to indicate acceptable fit values,^30^ and above .95 very good fit values.^31^ For the RMSEA, values below .06 are considered to indicate good fit. However, the RMSEA depends on model complexity. Therefore, the *P*-value for the test of close fit is also given, which tests the alternative hypothesis that the RMSEA is larger than .05. To indicate close fit, *P*-values should be larger than .05.^32^

## Results

Table 1 shows the study variables’ scales, means, standard deviations, Cronbach's alphas, and Pearson correlations. These results show that on average, burnout symptoms and coping self-efficacy were relatively stable variables throughout the years of measurement. Moreover, burnout symptoms and coping self-efficacy were moderately correlated in each of the years.

**Table 1.** Descriptive statistics (scale, mean, standard deviation, Cronbach's alpha, and Pearson correlations).

	Range	Mean	SD	n items	α	1	2	3	4	5
1. T1 Coping self-efficacy	1-5	3.93	0.42	10	.85					
2. T1 Burnout symptoms	1-7	2.13	0.89	10	.87	−.25*				
3. T2 Coping self-efficacy	1-5	3.93	0.47	10	.89	.69***	−.38***			
4. T2 Burnout symptoms	1-7	2.12	0.91	10	.89	−.26*	.76***	−.53***		
5. T3 Coping self-efficacy	1-5	3.97	0.46	10	.89	.67***	−.25*	.72***	−.28**	
6. T3 Burnout symptoms	1-7	2.04	0.96	10	.92	−.20*	.66***	−.51***	.77***	−.41***

*Note*: T = time. N = 95. * *p* < .05. ** *p* < .01. *** *p* < .001.

The empirical model showed satisfactory fit with the data (χ^2^ (*df *= 4) = 10.49, RMSEA = .13 (pclose = .01), CFI = .98, NFI = .97, TLI = .93). CFI, NFI, and TLI indicate adequate to good fit. Although the RMSEA was larger than .07, the *P*-value was below .05, indicating near satisfactory fit.

**Figure 2.**
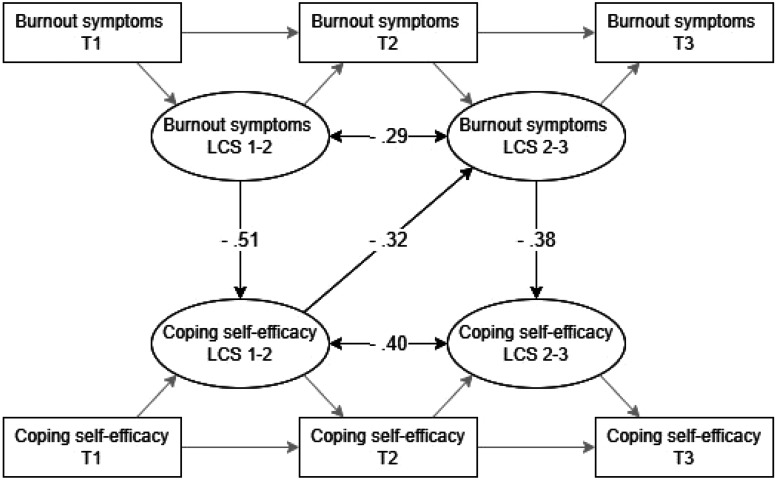
LCS model with results, presented in β-coefficients. A solid black arrow indicates a significant relationship as described by our hypotheses. Double arrows represent the significant autoregression effects. T = time, LCS 1-2 = Latent Change Score from time 1 to time 2, LCS 2-3 = Latent Change Score from time 2 to time 3.

Figure 2 presents the paths in the model and the corresponding regression coefficients. Findings showed that changes in burnout symptoms were negatively related to changes in coping self-efficacy in the same year (β = −.51, *P *< .001 and β = −.38, *P *< .001). That is, an increase in burnout symptoms was related to a decrease in coping self-efficacy within both one-year periods. Hypothesis 1 was supported. Furthermore, our results showed that a decrease in coping self-efficacy is related to an increase in burnout symptoms the following year (β = −.32, *P *< .01), supporting Hypothesis 2.

## Discussion

This study applied LCS modeling to analyse whether and how (contemporary and/or lagged) within-person changes in burnout symptoms are related to within-person changes in coping self-efficacy. Hypothesis 1 dealt with the effect of changes in burnout symptoms on changes in coping self-efficacy within the same 1-year period. In line with the theory on efficacy beliefs and how they are established,^5^ changes in burnout symptoms were negatively related to changes in coping self-efficacy. Hypothesis 1 was supported in two consecutive (approximately) 1-year periods, that is, police officers who experienced an increase in burnout symptoms during a 1-year period demonstrated a drop in coping self-efficacy during this same year. This finding confirms previous research on the negative relationship between burnout symptoms and coping self-efficacy, in that burnout is a complex negative affective state that can diminish self-appraisal and self-efficacy.^5,7^

Hypothesis 2 concerned the lagged effect of changes in coping self-efficacy during a 1-year period on changes in burnout symptoms during the following 1-year period. In line with the JD-R model,^14,15,23^ changes in coping self-efficacy in one year were negatively associated with changes in burnout symptoms the following year. Hypothesis 2 was supported, which emphasizes the buffering role of coping self-efficacy in preventing or reducing burnout symptoms.^3^

These findings indicate that a depletion of resources within individuals may occur and that this depletion can result in a loss cycle of resources, especially when initial burnout symptoms increase. Furthermore, we can conclude that resource depletion may pose a realistic risk for further burnout prevention in the following year.^cf^^33^

### Theoretical Implications

The results reinforce the buffering principle of the JD-R model in the sense that coping self-efficacy (a personal resource) has a buffering effect for burnout symptoms the following year.^14^ This is in line with results of previous studies.^1,3,15–17^ Also, they corroborate earlier research on coping resources and efficacy beliefs,^34^ showing that police officers who experienced an increase in coping self-efficacy also experience a decrease in burnout symptoms. Furthermore, it provides support for the COR theory, showing that personal resources reduce a health impairment process.^13^

In this study, we combined the buffering hypothesis (JD-R model) with the depletion hypothesis (efficacy beliefs). Together, the confirmed hypotheses imply that a loss cycle of resources can occur, supporting a basic premise of COR theory.^4,13^ Increasing burnout symptoms during the first year were associated with decreasing coping self-efficacy. These decreasing resources were related to increasing burnout symptoms, which again were related to decreasing self-efficacy. This suggests that stressful situations and increases in stress lead to having to allocate and thus deplete resources, which in turn can hinder these resources from being invested for future burnout prevention. This corroborates the results from earlier research that have included coping resources as an outcome of exhaustion^7^ and burnout symptoms.^3^

This study extends the previous research and emphasizes the complex dynamical nature of stress and coping resources. That is, stress and depletion of resources are parts of a dynamic process. The focus on within-person changes and dynamic processes instead of between-person designs and fixed levels of burnout or coping self-efficacy does more justice to the principles of COR theory and loss cycle. This provided us a chance to gain more substantiated insights into these complex dynamical processes.

Moreover, in contrast to a positive gain cycle of resources, efficacy beliefs, and engagement that Llorens and colleagues focused on,^6^ we focused on the negative loss cycle. That is, as an extension of the study by Llorens-Gumbau and Salanova-Soria who showed that burnout symptoms can lead to a decrease in self-efficacy,^7^ we showed that this in turn led to more burnout symptoms for individuals within the same study. Previous research had not yet demonstrated that loss cycles of resources exist in this way, especially using LCS modeling. In the end, the results emphasize the necessity of researching loss cycles and of monitoring individual wellbeing, stress, and preservation of resources for wellbeing.

### Practical Implications

This study highlights the importance of efficacy beliefs and availability of resources for police officers. Our findings indicate that burnout can have a negative impact on coping resources, while these resources are important to prevent future burnout. Therefore, police organizations and police officers individually would benefit from monitoring changes in burnout symptoms and coping resources over time. Moreover, individuals who experience a limited amount of chronic strain or burnout symptoms could still benefit from increasing their resources to reduce the risk of future burnout. For individuals who already experience stress or burnout symptoms this may not be as simple, as they may lack the energy and resources in the first place to be able to behave proactively and create a (more) positive self-image. For these individuals it is of additional value to monitor these issues. This can enable them and their organizations to detect at an early stage when a loss cycle is instigated, so that timely support and interventions can be deployed.

### Limitations and Directions for Future Research

One limitation of this study is that we used self-report measures for burnout and coping self-efficacy. Although, the longitudinal nature of the study (ie, measuring three years in a row) reduces the likelihood of common method bias. It is important to recognize self-report measures as a limitation of research and in future studies consider more objective data (eg, take into account occupational health physicians’ statements on prevalence of burnout symptoms within an organization).

Also, the average level of self-reported burnout symptoms is low. This is likely due to selection bias of a relatively healthy working sample of police officers. Future researchers could compare the results with individuals who were previously diagnosed with (clinical) burnout and who may currently be reintegrating or have recently reintegrated.

Another limitation lies in the lag of the measurements. Evidence was found for a depletion mechanism that occurred simultaneously, that is, within the same unit of time. In this study, this period was approximately one year, which is quite broad and may have reduced the chances of finding a depletion effect of one period onto the next. Therefore, future research could investigate shorter lags between the measurement to establish whether a depletion over a shorter time period also occurs. Furthermore, future research might also deal with the existence of more complex mechanisms in the process under study, by encompassing multiple waves, adopting a time series approach with at least four or more waves in a ‘shortitudinal’ design, and perhaps by incorporating other concepts that may affect coping self-efficacy or resources in general such as reorganizations, job insecurity, or poor work-life balance.

## Conclusion

Using LCS modeling to investigate within-person changes, we found that increases in burnout are related to decreases in coping self-efficacy during the same year. Additionally, the buffering role of coping self-efficacy for burnout in the following year was confirmed. This study supports that a depletion, or loss cycle of resources may occur. Therefore, it is important to be aware and monitor for the presence and absence of burnout and resources, to maintain or promote future employability.
